# Imaging Assessment of Endothelial Function: An Index of Cardiovascular Health

**DOI:** 10.3389/fcvm.2022.778762

**Published:** 2022-04-15

**Authors:** Anum S. Minhas, Erin Goerlich, Mary C. Corretti, Armin Arbab-Zadeh, Sebastian Kelle, Thorsten Leucker, Amir Lerman, Allison G. Hays

**Affiliations:** ^1^Division of Cardiology, Department of Medicine, The Johns Hopkins University School of Medicine, Baltimore, MD, United States; ^2^Department of Epidemiology, Johns Hopkins Bloomberg School of Public Health, Baltimore, MD, United States; ^3^Department of Internal Medicine and Cardiology, German Heart Center Berlin, Berlin, Germany; ^4^Division of Ischemic Heart Disease and Critical Care, Department of Cardiovascular Medicine, Mayo Clinic, Rochester, MN, United States

**Keywords:** coronary MRI, endothelial function, CAD, CMR, vascular disease

## Abstract

Endothelial dysfunction is a key early mechanism in a variety of cardiovascular diseases and can be observed in larger conduit arteries as well as smaller resistance vessels (microvascular dysfunction). The presence of endothelial dysfunction is a strong prognosticator for cardiovascular events and mortality, and assessment of endothelial function can aid in selecting therapies and testing their response. While the gold standard method of measuring coronary endothelial function remains invasive angiography, several non-invasive imaging techniques have emerged for investigating both coronary and peripheral endothelial function. In this review, we will explore and summarize the current invasive and non-invasive modalities available for endothelial function assessment for clinical and research use, and discuss the strengths, limitations and future applications of each technique.

## Introduction

Despite declines in cardiovascular disease (CVD) mortality rates over the past few decades, CVD still remains the leading cause of morbidity and mortality in the United States (1). Endothelial dysfunction contributes to atherosclerosis development and progression, which may ultimately lead to plaque rupture and cardiovascular events. Although the vascular endothelium serves many important functions including maintaining vasomotor tone and barrier functions, the most readily detectable means to define endothelial pathology or dysfunction in humans is by quantifying vasomotor responses to endothelial dependent stressors. The development in recent years of imaging strategies to measure endothelial function of the coronary and peripheral vessels has provided insights into important contributors of coronary artery disease (CAD) and the vascular response to therapeutic intervention. In this review, we will briefly examine mechanisms relating endothelial function and atherosclerosis, review imaging strategies, both invasive and non-invasive, to quantify endothelial function of the coronary and peripheral circulation, and discuss recent insights from human endothelial function studies.

## Overview: The Vascular Endothelium

Dysfunction of the vascular endothelium is increasingly recognized as serving a prominent role in CVD pathology. The endothelium regulates vascular tone, smooth muscle cell proliferation, thrombosis, and leukocyte adhesion and platelet aggregation (2). Endothelial dysfunction, or alteration in normal function, often precedes the development of anatomic atherosclerotic disease progression and clinical manifestation. Examination of endothelial function can enhance risk stratification, improve early detection of disease and be used to assess the vascular response to therapeutic intervention (3).

Healthy endothelial cells respond to local and systemic factors by producing and releasing vasoactive molecules to maintain vascular tone, a balance between vasodilation and vasoconstriction (4). A defining feature of endothelium-dependent relaxation is the release of nitric oxide (NO), which diffuses to vascular smooth muscle cells and results in cGMP-mediated vasodilation (4). NO is released in response to a variety of signals, such as adenosine, serotonin, catecholamines, ischemia, and shear stress (5). Conversely, systemic inflammation and increased reactive oxygen species (ROS) tend to counter the effects of NO, and can result in chronic endothelial dysfunction (6). Cardiovascular risk factors such as hyperlipidemia, hypertension, and diabetes may result in dysregulation of endothelial nitric oxide synthase (eNOS) and ROS (7), leading to endothelial dysfunction, one of the earliest steps in the atherosclerotic disease process (8). Although dysfunctional endothelium is characterized by increased vascular inflammation, permeability and thrombosis, it is impaired vasodilation in response to stressors that increase NO that is the most readily measurable response in humans and detectable by imaging.

In the peripheral conduit vessels, endothelial function is typically evaluated in the brachial artery due to its accessibility, and measures can be performed invasively (forearm plethysmography) or non-invasively (brachial ultrasound for flow mediated dilation) by evaluating the vasomotor response to endothelial dependent stressors (9). Measuring endothelial function of the coronary arteries is more challenging but important as the clinical impact of coronary endothelial dysfunction is greater than other vascular beds. Coronary endothelial function (CEF) is typically examined through invasive measures during coronary angiography. Coronary arteries are prone to atherosclerosis and studying CEF provides new information about the heterogeneity of endothelial function and contributors to plaque formation in patients with, or at risk for coronary artery disease. However, the invasive measurement of CEF carries procedural risk and preclude studies in lower risk patients over time. Newer non-invasive measures of CEF including with magnetic resonance imaging (MRI) and positron emission tomography (PET) promise new insights into the pathophysiology of CVD in low risk and other populations not undergoing invasive angiography and can assess response to therapy. Finally, microcirculatory assessment of smaller vessels, comprised of pre-arterioles, arterioles, capillaries and venules, investigates endothelial function in vascular resistance, which mediates blood pressure and blood flow. The measure of endothelial function of the larger (conduit) or smaller (microvessels) provides important and complementary information which can help gauge CV risk and provide prognostic information for patients (10).

## Techniques to Measure Endothelial Function in Humans: Invasive Coronary Endothelial Function Assessment

**FIGURE 1:**
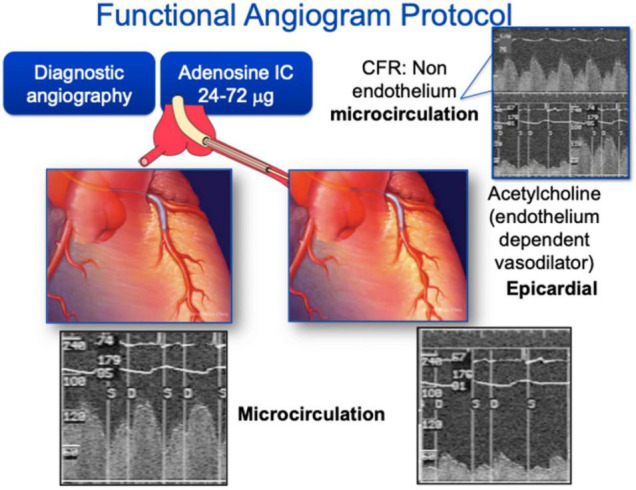
Coronary angiography for endothelial function assessment. Coronary angiography can be used for both epicardial and microvascular function assessment. Typically, acetylcholine is used as the endothelium dependent vasodilator for epicardial coronaries and adenosine is used for microcirculation assessment.

The measurement of human endothelial function primarily focuses on vasoreactivity testing, as this is the most clinically demonstrable function of the vascular endothelium (11). Coronary endothelial dysfunction predicts cardiovascular events and remains the most important vascular bed studied in vasoreactivity (10, 12, 13). The gold standard for coronary endothelial functional assessment is via invasive quantitative angiography to detect luminal changes in response to vasoactive stimuli, either pharmacologic or physiologic, that increase the endothelial release of NO (Figure 1) (14, 15).

Coronary angiography for epicardial arterial dimension measurement is often performed with intracoronary infusion of acetylcholine. Acetylcholine is an endothelial-dependent vasodilator that is suitable for intracoronary infusion and is the most commonly used drug for the purposes of invasive vasomotor testing. Healthy endothelium should result in coronary arterial vasodilation and increased blood flow (by >50%) in response to low dose acetylcholine, while dysfunctional endothelium may lead to diminished blood flow response and even to paradoxical vasoconstriction. At higher doses, acetylcholine can result in constriction of small arteries via direct effect on smooth muscle cells, and may be used to evaluate microvascular function (16). Less commonly, other agents used in endothelial-dependent vasomotor testing have included bradykinin, papaverine and Substance P (17). In addition, adenosine has partial endothelial dependent effects (18). These vasoactive agents act on coronary microvasculature through vasodilation and increased flow, resulting in NO release and proximal coronary artery vasodilation, or flow-mediated dilation (FMD) (19, 20), permitting the study of epicardial endothelial function.

Coronary microvascular function can be studied invasively by measuring coronary blood flow changes and thereby coronary flow reserve using a Doppler wire. Generally, this is accomplished by placement of a Doppler-tipped guide wire into the coronary artery of interest, whereby continuous blood flow velocity is measured both at baseline and during intracoronary infusion of vasoactive substances (acetylcholine, adenosine, or papaverine) through the guiding catheter (17, 21, 22).

Further, invasive CEF assessment can also be performed by cold pressor testing (CPT) or exercise testing, both endothelial-dependent stressors (23). Exercise stress testing can be performed while supine using a bicycle ergometer with concurrent hemodynamic monitoring (24). Healthy coronary arteries dilate in response to these stressors, while paradoxical vasoconstriction occurs in diseased coronary arteries, suggesting underlying endothelial dysfunction.

Endothelial dysfunction diagnosed by invasive methods has been reported in several cardiometabolic disease states and is associated with future atherosclerosis and other adverse outcomes (13, 25–27). These techniques have also been used in the assessment of endothelial dysfunction reversal with treatment therapies (28). The advantages of catheter-based methods of coronary endothelial assessment include the precision and accuracy of results obtained using this gold standard of testing, particularly in comparison to techniques that rely on surrogate measures of coronary arterial function (17). With this approach, however, come the limitations of an invasive procedure with intra-arterial injection of vasoactive medications that can have systemic adverse effects, along with exposure to radiation and contrast. Given these risks, repeat evaluation is often not performed. Invasive techniques are therefore largely limited to patients undergoing coronary angiography for clinical reasons. Additionally, in patients with CAD, vessel area measurements may be limited in coronary segments with atherosclerosis.

## Non-Invasive Evaluation of Epicardial Coronary Endothelial Function

### Magnetic Resonance Imaging for Assessment of Coronary Endothelial Function

Magnetic resonance imaging provides a reproducible and safe means to measure CEF non-invasively without contrast and with high spatial resolution. In addition, MRI offers the ability to quantify coronary blood flow velocity and determine blood flow, important in the assessment of microvascular endothelial vasoreactivity, as well as measures of vessel wall remodeling, important in the detection of early atherosclerosis. MR measures of coronary area and blood flow velocity have been validated and compared to invasive measures using quantitative coronary angiography with Doppler techniques in response to stress (29–31). However, MRI has not been exploited to investigate coronary endothelial-dependent vasomotor responses in healthy and diseased states until more recently.

**FIGURE 2:**
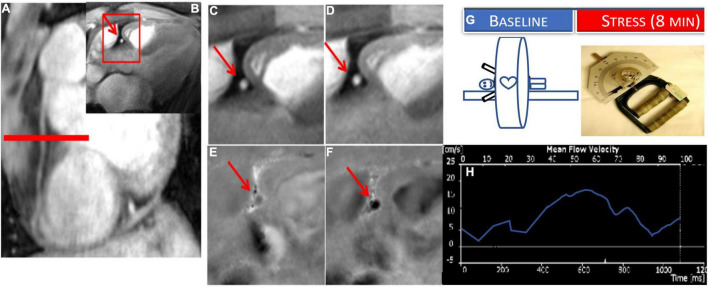
Example of coronary endothelial function (CEF) testing using non-contrast MRI with isometric handgrip exercise (IHE). Scout MRI **(A)** and cross-sectional cine **(B–D)** and phase-contrast images **(E,F)** in a healthy subject showing RCA in cross-section (red arrow). In the expanded inset sections, coronary area increases from rest **(C)** to IHE **(D)** and velocity and flow increase from rest **(E)** to stress **(F)** (note that increased darkness represents increased signal and thus velocity down through the imaging plane). **(G)** Stress MRI protocol for CEF measures for endpoints: change in coronary cross sectional area and blood flow velocity (%) from baseline to stress (continuous IHE for 5–8 min). **(H)** Example coronary flow velocity curve of RCA.

To measure CEF non-invasively, coronary MRI has been combined with isometric handgrip exercise (IHE), a known endothelial-dependent stressor to quantify IHE-induced coronary cross sectional area and blood flow change as quantitative measures of CEF (32). Using these MRI-IHE methods, initial studies showed impaired CEF in patients with CAD (32, 33) and separately in people living with HIV compared to risk factor matched control participants (34–36). MR images were taken perpendicular to a proximal or middle straight segment of the coronary artery best identified on scout images (Figure 2) and all quantifications were performed during a period of least cardiac motion as previously described (32, 37, 38). Both anatomical (cross sectional area) and velocity-encoded (for coronary velocity and flow) images were quantified at baseline and during approximately 5 min of continuous isometric handgrip exercise while under direct supervision to ensure compliance. In addition, endothelial independent coronary vasoreactivity was assessed in a subset of healthy volunteers and CAD patients who additionally received sublingual nitroglycerin, and imaging was repeated (32). Moreover, the degree of coronary artery luminal stenosis in a given CAD patient was compared to local CEF within the same segment. In this initial study, normal, physiologic coronary vasodilation and increased coronary velocity and blood flow were observed in healthy subjects in response to handgrip, but not in CAD patients. Nitroglycerin, an endothelial- independent stressor induced normal vasodilation in patients with CAD, indicating preservation of vascular smooth muscle relaxation in the same segments where endothelial function was abnormal (32). Importantly, local CEF was more severely impaired in areas with significant luminal stenosis and early coronary wall thickening than that in minimally diseased vessels (32, 33). Furthermore, reproducibility (including intra-interobserver and interscan) on the same day and over time (8 weeks) was robust, important for designing future intervention studies using this technique (32, 39). Therefore, these MRI methods to non-invasively and reproducibly characterize CEF provide an opportunity to allow the monitoring of inventions aimed at an early stage of coronary disease. The main limitation of the technique is lack of widespread availability and that the 2D approach does not permit CEF measurements of the entire coronary tree. Finally, because the protocol involves serial breath holds, the study may be difficult in sicker patients with respiratory problems.

### Vascular Insights of Coronary Endothelial Function Studies

Important for any new study measuring endothelial function is to demonstrate that the vasoreactive response being measured truly reflects NO-mediated endothelial function. The normal coronary vasoreactive response to IHE detected by MRI was quantified before and during the infusion of the NO synthase inhibitor, NG-monomethyl-L-arginine (L-NMMA), to determine if the coronary response to IHE is NO-mediated, the defining feature of endothelial function (39). In this study, L-NMMA infusion blocked the normal coronary vasodilatory response and coronary blood flow increase with IHE in healthy participants, demonstrating that IHE is a primarily NO-dependent endothelial coronary stressor that can be combined with MRI to measure CEF. In addition, similar approaches were employed to quantify endothelial function of the internal mammary artery (IMA), a systemic vessel that rarely develops atherosclerosis, is often used as a coronary artery bypass graft, and has been previously used to study systemic endothelial function (38). These initial studies showed that the IMA response to IHE was NO-dependent and reproducible, was impaired in patients with CAD compared to healthy subjects and differed from the endothelial response of the coronary arteries in a given patient. In summary, MRI promises a non-invasive assessment of coronary vascular health that can be safely applied to low- and medium risk populations without the risks of invasive angiography.

## Positron Emission Tomography/Computed Tomography for Assessment of Coronary Endothelial Function

Nuclear imaging methods can be used to evaluate myocardial blood flow and response to endothelial-dependent stressors. PET can be used to estimate coronary flow reserve and myocardial regional perfusion using intravenously injected tracers (^15^Oxygen-labeled water, ^13^Nitrogen-ammonia, and ^82^Rubidium), and studies have revealed abnormalities in endothelial function prior to visible atherosclerosis on angiography (40, 41). These techniques have been successfully combined with CPT to assess CEF. CPT protocols typically involve immersion of the subject’s hand or foot into an ice bath at 2°C for at least 1 min prior to radioactive tracer injection and PET scan (42). CPT functions to increase myocardial oxygen demand via sympathetic activation, which should cause vasodilation and an endothelial-dependent increase in coronary blood flow in healthy subjects (43). Using these principles, cardiac PET during CPT has been shown to reflect epicardial vasomotor dysfunction in subjects at high risk for CAD (44). Abnormalities in myocardial blood flow on PET, regardless of concurrent CAD, appear to confer an increased relative risk of death and heart failure (42, 45). It is important to recognize that myocardial blood flow is affected by epicardial coronary vasomotor tone and microvascular function, making it challenging by PET imaging alone to determine whether changes in flow are related to conduit or resistance vessels (41).

The addition of computed tomography (CT) to PET can further enhance the sensitivity for atherosclerosis detection (46). A hybrid PET/CT approach has the ability to quantify changes in coronary cross-sectional area in response to stress, global, and relative myocardial perfusion, left ventricular functional performance, and coronary calcium score. This non-invasive tool for assessing coronary vascular health may represent a clinically relevant evaluation that can be performed in early disease or to predict downstream risk, however, its use has been primarily research-related (47).

## Non-Invasive Evaluation of Myocardial Blood Flow Reserve as a Measure of Coronary Microvascular Function

### Positron Emission Tomography

**FIGURE 3:**
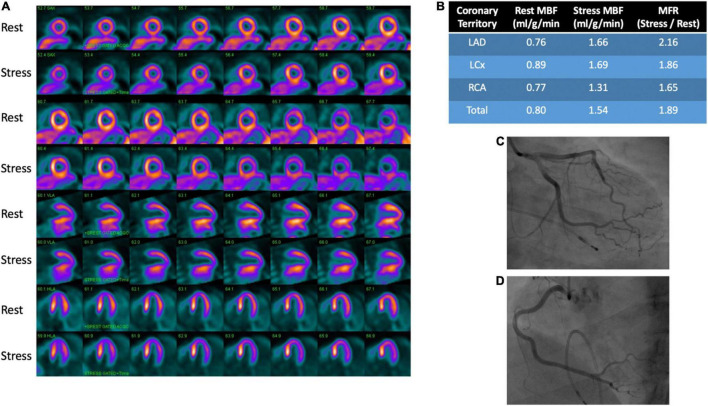
PET rest/stress images and coronary angiography in a patient with microvascular dysfunction. **(A)** Perfusion images demonstrate no evidence of stress (regadenoson)-induced myocardial ischemia. **(B)** Provides quantitative myocardial perfusion analysis with an overall reduced coronary flow reserve of 1.89, indicative of mild diffuse microvascular disease. The functional analysis for this patient showed normal wall motion. No obstructive coronary artery disease was seen on angiography of the left anterior descending **(C)**, left circumflex **(C)** and right coronary **(D)** arteries.

Among the currently available non-invasive methods for measuring myocardial blood flow and myocardial flow reserve with stress, PET is the most well studied and validated test (48). Images are obtained at rest and vasodilator-induced stress following injection of a radiotracer. Post-processing of images is then performed to quantify regional and global myocardial blood flow (ml/min/g of myocardium) (45, 49). Myocardial flow reserve (MFR) is calculated as the ratio of stress to rest myocardial blood flow (MBF). MBF is affected by myocardial oxygen demand, contractility, heart rate, blood pressure and preload, resulting in a reported resting MBF ranging from 0.4 to 1.4 ml/g/min (50). Typically, MFR < 2.0 is considered abnormal and consistent with microvascular dysfunction in the absence of significance epicardial disease as changes in MFR can be due to epicardial and/or microvascular changes in blood flow (50). A representative image is provided showing rest and stress images with PET in a patient with microvascular dysfunction and no CAD on invasive angiography (Figure 3).

Multiple studies have evaluated the prognostic implications of MFR by PET. Studies have demonstrated that dysfunction seen on PET can identify individuals at high risk for major adverse cardiac events and cardiovascular death in those with and without obstructive CAD (51–53). Moreover, PET has been shown to reclassify risk in about one third of patients when compared to only traditional cardiovascular risk factors (54). The benefits of PET in prognosticating cardiac death may be particularly evident in specific groups such as those with cardiometabolic diseases (55, 56). Notably, abnormal MFR on PET has also been shown to be predictive of hospitalizations for heart failure in patients with heart failure with preserved ejection fraction (57). Despite several studies enhancing risk assessment using PET, there are limited studies using PET measures to evaluate therapeutic interventions and response, likely due to concerns about radiation exposure. Prior studies using PET imaging have examined the therapeutic response to statins and bariatric surgery (45, 58, 59).

Ultimately, the advantages of dynamic PET myocardial imaging include validation by microsphere blood flow studies in preclinical animal models and human studies (60, 61). PET also offers better spatial resolution and lower radiation exposure compared to single-photon-emission-computed-tomography (SPECT) perfusion (60). However, PET imaging is associated with high cost, limited radiotracer availability and advanced equipment, which can be a limitation to routine and widespread use.

## Cardiovascular Magnetic Resonance Perfusion Imaging

Non-invasive assessment of impaired myocardial blood flow, which contributes to ischemia in patients with CAD and cardiomyopathy, can be performed using stress perfusion cardiovascular magnetic resonance (CMR), which may be especially helpful for serial examinations evaluating treatment success (62). Stress perfusion CMR, distinct from coronary vasoreactivity approaches mentioned above, typically uses vasodilator stress (i.e., adenosine) to detect macrovascular (i.e., coronary stenosis) and microvascular differences in myocardial blood flow in response to stress. Recently, studies have employed fully quantitative stress myocardial perfusion techniques in patients with no obstructive CAD and detected reduced myocardial perfusion reserve, not explained by cardiac hypertrophy or fibrosis (63). The ability of CMR to study ventricular function/structure and fibrosis make it well-suited to be used in combination with stress perfusion techniques, especially in patients with left ventricular hypertrophy.

Stress perfusion CMR techniques have also been employed to evaluate patients at risk for microvascular dysfunction. Clinical guidelines have recently added microvascular dysfunction to epicardial stenosis and epicardial coronary spasm as one of the mechanisms of myocardial ischemia in patients with CAD (64). One study used stress CMR techniques and showed that myocardial perfusion reserve index was impaired in women with no obstructive CAD on coronary angiography, reflecting microvascular dysfunction compared to reference controls (65). A randomized trial in this setting showed that medical therapy with ranolazine improved angina and reduced ischemic burden in woman with myocardial ischemia detected by stress CMR in the absence of obstructive CAD, suggesting a possible use of MRI for therapeutic assessment (66). Additionally, in patients with infiltrative heart disease such as amyloidosis, it has been demonstrated that impaired myocardial perfusion is related to abnormalities in myocardial structure and function not only at stress, but also at rest (67). Taken together, studies support the use of stress perfusion CMR to investigate myocardial perfusion reserve, which reflects microvascular dysfunction in the absence of CAD. In addition, stress CMR has already demonstrated high prognostic value and cost-effectiveness compared to invasive strategies (68, 69). While classically, limitations of this technique included the need for highly specialized equipment and providers, recent technical developments now allow quantitative and fully automated assessment of myocardial ischemia using stress CMR, which may enable the broad use of this modality outside of specialized centers (70).

## Computed Tomography Angiography

**FIGURE 4:**
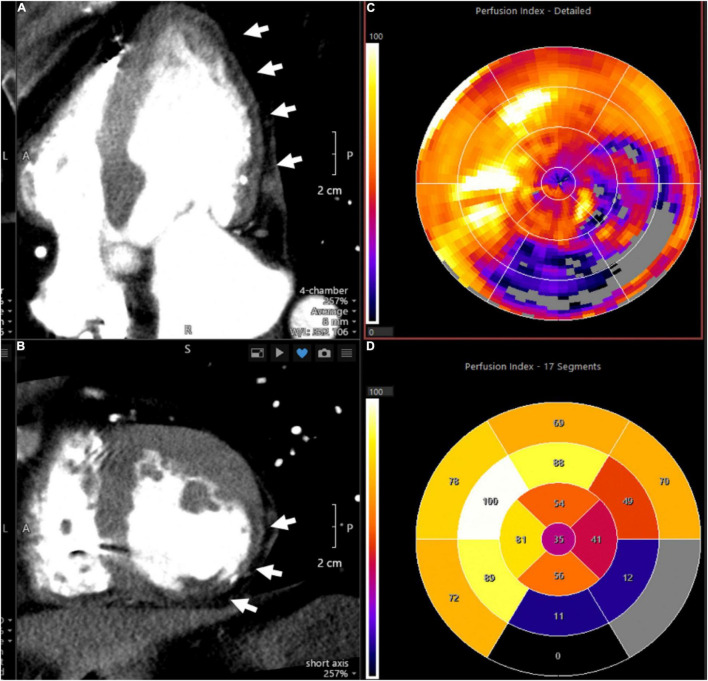
Representative CT perfusion images and polar plots. Images demonstrate severe myocardial perfusion abnormalities in the lateral and posterolateral walls in a patient with history of myocardial infarction. **(A)** Depicts a cardiac four-chamber view with arrows pointing to hypodense areas in the subendocardial and mid myocardial levels, representing perfusion defects. In addition, thinning of the myocardium is consistent with prior infarct. **(B)** Provides a cross-sectional assessment of the same case. **(C)** (Polar plot) shows the corresponding perfusion indices, with the affected myocardial segments provided in **(D)**.

The homogeneity of myocardial perfusion can be readily assessed by its uptake of iodine contrast medium and its associated X-ray attenuation. George et al. demonstrated that myocardial perfusion can be quantified using CT and that reversible perfusion defects can be identified after vasodilator challenge analogous to nuclear imaging techniques (71). CT scanning is performed using injection of an iodinated contrast agent with prospective electrocardiographic gating. Microvascular function may be assessed by determining MBF at rest and after vasodilator challenge with abnormal flow reserve typically defined as a ratio of <2.0 (60). In the absence of obstructive CAD and local myocardial perfusion defects, reduced MFR can be attributed to microvascular dysfunction. Figure 4 shows an imaging example of a patient with a severe myocardial perfusion defect in the lateral and posterolateral walls post infarct.

Advantages of CT include faster image acquisition than with nuclear techniques and markedly superior spatial resolution. Directly compared to nuclear myocardial perfusion imaging using exercise or vasodilator challenge, CT myocardial perfusion yields at least equivalent accuracy for identifying patients with CAD (72). Another major advantage of cardiac CT is the assessment of both coronary arterial anatomy and myocardium. Using contemporary technology, rest-vasodilator CT for coronary angiography and myocardial perfusion imaging can be performed with radiation doses lower than standard nuclear perfusion imaging using SPECT, though requiring two contrast applications of approximately 60 ml each (72). Determining MBF and coronary flow reserve by CT myocardial perfusion imaging is possible using dynamic imaging, i.e., continued imaging over several cardiac cycles (73).

Application of dynamic CT imaging had been hindered by high associated radiation exposure to the patient but new protocols have been developed using lower tube settings which have reduced radiation to levels similar to that by conventional rest-vasodilator myocardial perfusion protocols (73). Comparison to PET revealed high accuracy of dynamic CT for detecting abnormal MBF using a mean radiation dose of 8.4 mSv (74). Further radiation dose reductions are feasible using intermittent instead of continuous scanning, thus overcoming one of the major limitations of dynamic CT perfusion imaging and opening the possibility of comprehensive coronary arterial and myocardial assessment.

## Peripheral Endothelial Function Assessment

### Brachial Artery Flow Mediated Dilatation

**FIGURE 5:**
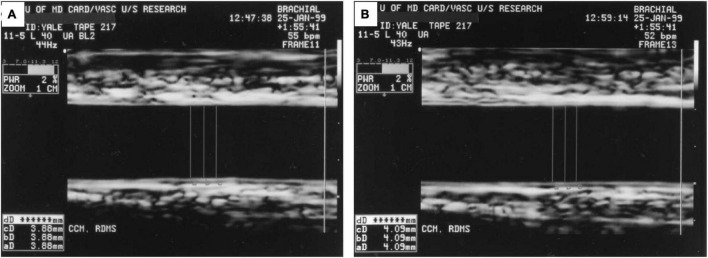
Ultrasound Images Demonstrating Brachial Flow-Mediated Dilatation. **(A)** Shows the brachial artery at rest with arterial diameter of 3.88 mm. **(B)** Shows the artery 1 min after hyperemic stimulus with arterial diameter of 4.09 mm. Figure reproduced with permission from Corretti et al. (9) copyright JACC (Elsevier).

In the early 1990s, high-resolution B-mode ultrasound and Doppler emerged as a non-invasive tool to measure brachial artery diameter and flow changes in response to vasomotor stimuli in research investigations of endothelial function, and remain as such currently (9, 75). Specifically, flow-mediated vasodilatation (FMD) of the brachial artery (or forearm radial artery) measures a focal segment of the artery to dilate in response to NO release induced by a 5 min blood pressure cuff occlusion and release (hyperemic stimulus). Oral nitroglycerin is typically used as the non-endothelium dependent vasoactive stimulus. Calculation of the % FMD is the percent change in arterial diameter post-stimulus compared to the baseline diameter, measured manually or with edge-detection software (76). Doppler velocity of the artery is also acquired at baseline, and upon immediate and 2 min post cuff release. Baseline and hyperemic blood flow are calculated from the time-averaged pulsed Doppler spectral trace time-velocity integral (NOVA Medical School) from the onset of one waveform to the beginning of the next waveform. A representative image is shown in Figure 5 (9). Over the course of time, there have been some modifications of the technical method and exam protocol, but studies relying on this technique provide insight into endothelial function at the imaging site, the time course of diameter changes and flow, and the role of distal microvascular physiology (75, 77).

Advantages of the FMD technique include relative cost-effectiveness, easy access, availability, and validated digital software for more automated analyses. In addition there is robust reproducibility in experienced labs and importantly, strong evidence that endothelial dysfunction measured with FMD predicts cardiovascular events (78). However, optimal acquisition of the vessel images and Doppler in a time-sensitive manner is technically challenging, with a significant learning curve to achieve and maintain high-quality, consistent performance and reproducibility in data acquisition and interpretation. Differences in methodological technique and exam protocols also limit the comparability, accuracy, validity, and reproducibility. Nevertheless, brachial FMD methods provide a validated non-invasive assessment of endothelial function.

### Venous and Arterial Plethysmography

Venous occlusion plethysmography is an invasive, extensively used research technique to study human vascular physiology and pharmacology *in vivo*. The technique indirectly measures microvascular function as forearm blood flow in response to an intra-arterial infusion of a vasoactive substance such as acetylcholine, adenosine, or nitroglycerin into either the brachial or radial artery, or alternatively to reactive hyperemia induced by increased shear stress. The contralateral arm is used as the control, and the results are expressed as the ratio of the changes in flow measured in both arms (79). Training is essential to ensure standardization and quality control. The technique is validated, reliable, and highly reproducible (79). However, its invasive nature precludes application for routine clinical use. Additionally, the various anatomic, physiologic and technical factors involved with venous plethysmography limit its application to study changes between individuals, groups or in large populations. A limitation to plethysmography and applanation tomography include lack of standardization. Nonetheless, it remains a valuable research tool to evaluate the pathologic mechanisms underlying endothelial dysfunction, the effect of various therapeutic interventions and risk factor modifications.

Similarly, finger plethysmography (peripheral arterial/amplitude tonometry) can be used to assess peripheral endothelial function in the digital microvasculature (75, 80). Pulse amplitude tonometry (commercially available as Endo-PAT2000 (Pulse Arterial Tone), Itamar Medical) is an FDA approved product that records pulse amplitude in the individual’s fingertip at rest and during reactive hyperemia (81). Hyperemia is induced by occluding blood flow through the brachial artery for 5 min using an inflatable cuff. Hyperemia in the fingertip increases the pulse amplitude. Proprietary software is applied to obtain the net response is expressed as the reactive hyperemia pulse amplitude tonometry index (RHI), considered a marker of endothelial function. The endothelium-mediated change in the PAT signal, elicited by the downstream hyperemic response, is calculated automatically by the system. A PAT ratio is then created using the post and pre occlusion values normalized to measurements from the contralateral arm (control). Importantly, studies have shown that peripheral microvascular dysfunction predicts future cardiovascular events (82, 83).

### Endothelial Function in the Coronary vs. Peripheral Circulation

Although abnormal systemic and coronary endothelial function are predictors of cardiovascular events, vasoreactivity across different vascular beds are not always closely associated. Studies comparing to coronary systemic endothelial function have shown that the correlation between the two may be modest (38, 84). Further, other studies have shown that endothelial dysfunction is not always uniform across vascular regions or even within the coronary tree of the same individual (32, 38). These regional differences in endothelial function may be due differences in local shear, downstream resistance vessels, neurohormonal regulation or propensity to develop atherosclerosis and plaque rupture. Taken together, endothelial function measures of different vascular beds may provide complementary information, each with unique strengths and limitations (Table 1). However, further studies are need to elucidate the relative role of endothelial measures in different vessels.

**TABLE 1:** Comparison of the invasive and non-invasive methods for assessing endothelial function.

Modality	Strengths	Limitations
Coronary angiography	• Gold standard method • Direct visualization and quantitation of endothelial function • Able to assess dose-response • Precise and accurate results	• Invasive • Expensive • Vasoactive medications can have systemic effects • Largely limited to clinical studies
Brachial artery flow mediated dilatation	• Non-invasive • Cost-effective • Validated software for automated analyses • Well correlated with coronary endothelial function	• Operator dependent • Technically challenging to obtain optimal images • Variable measurements, which limit comparability and reproducibility
Forearm plethysmography/Applanation tonometry	• Minimal training required • Inexpensive • Portable • Well tolerated • Can provide indirect information on the structure of small resistance arteries	• No clear cutoff values • Used mostly for mechanistic research studies • Limited reproducibility • Requires specialized training for standardization • Findings may not reflect endothelial function only
Venous occlusion plethysmography	• Validated technique • Reproducible • Easier to access than coronary arteries	• Invasive • Limited ability to compare application between individuals or groups
Positron emission tomography	• Well-validated in animal and human studies • Automated software for quantitative analysis	• Radiation exposure • Expensive • Lack of easy access
Computed tomography	• Good spatial resolution • Relatively cost-effective • Fast image acquisition	• Radiation exposure • Image may be compromised by increased heart rate • Calcium related beam hardening may result in artifacts
Magnetic resonance imaging	• High spatial and temporal resolution • No ionizing radiation • Cardiac structure and function assessment included	• Limited availability • Expensive • Long study length • Limited use in patients with arrhythmias, claustrophobia or implanted devices

## Clinical Studies and Applications

Both established and newer cardiovascular risk factors can adversely affect endothelial function, including obesity, diabetes, smoking, and inflammation/oxidative stress (7, 8). To this end, the measurement of endothelial vasoreactivity serves as an index of the sum total effects of environmental and genetic factors on the vasculature. Furthermore endothelial dysfunction is a marker for subclinical disease, an independent predictor of adverse cardiovascular events, and a potential target for medical interventions (78, 82). One study using PET showed that cigarette smokers have reduced MFR, with improvement seen with smoking cessation (85). Similarly, initiation of antihypertensives can result in improved endothelial function in patients with hypertension (86). Obese patients were reported to demonstrate impaired MBF with improvement after bariatric surgery (59). In addition, MFR is reduced in patients with diabetes, with some suggestion that endothelial function (measured by FMD) may improve with dapagliflozin (55, 87).

Recently, coronary microvascular dysfunction has been implicated in multiple disease processes including microvascular angina, a common encountered disorder which can lead to ischemia or myocardial infarction, even in the absence of obstructive coronary artery disease (60). Microvascular angina due to ischemia with non-obstructive coronary arteries (INOCA) can be challenging to diagnose, with a heterogenous approach to patients and many knowledge gaps with regards to treatment. The CorMicA trial showed that guiding therapy by invasive provocative coronary testing in patients with INOCA identified to have microvascular dysfunction may be of clinical benefit (88). Recent methods using stress perfusion CMR are being employed in the CorCMR study to evaluate whether a non-invasive approach to assess coronary microvascular dysfunction in INOCA patients improves cardiovascular risk and anginal symptoms (89). The results of study may have important clinical implications in this patient population, where there is less evidence in terms of diagnostic testing and treatment. Furthermore, using these approaches to quantify coronary microvascular function may provide clinically meaningful information beyond what is possible using standard anatomic and ischemia assessment with the ultimate goal of improving patient outcomes. It is important to note that many of the techniques described in this review (PET, specialized CMR, and CT perfusion) are not yet widely available clinically, however, may play an important role in the evaluation of patients with INOCA and to test early therapies to justify larger clinical trials with hard end points.

Both coronary and systemic endothelial function measures have been used as endpoints in clinical intervention trials after the techniques were shown to be reproducible in the short and intermediate term (90). Studies using endothelial function as an endpoint enable the assessment of the vascular impact of emerging treatment strategies and can guide novel drug development, such as approaches to target oxidative stress or inflammation. Studies targeting the xanthine oxidase (XO) system, a significant source of vascular oxidative stress, or systemic inflammation using colchicine have used CEF testing as a surrogate imaging endpoint over time in randomized placebo-controlled clinical trials (90, 91). Recently, impaired CEF in people with HIV and dyslipidemia improved with short term treatment with the PCSK9 inhibitor, evolocumab, indicating that the MRI-CEF technique can detect rapid improvements in CEF in response to treatment (92). Therefore, this approach enables future studies focused on repeated CEF measures in healthy and lower risk populations over time.

## Conclusion

Endothelial dysfunction is now a well-established gauge of cardiovascular risk and predicts future adverse events. Recently, endothelial dysfunction has been implicated as a contributor to a variety of cardiovascular diseases including INOCA, stress cardiomyopathy, preeclampsia and heart failure with preserved ejection fraction among others (26, 93). We have summarized multiple methods that are available for probing coronary and peripheral endothelial, each with specific strengths and weaknesses, and different values for defining pathology (Table 2). Currently available endothelial testing methods are helpful for mechanistic understanding of disease and for risk stratification and prognostication. It is important to recognize, though, that using a pharmacological stressor for imaging to assess endothelial function will often detect function in response to a combination of endothelial and non-endothelial dependent mechanisms, and depending on the stressor and imaging modality, this should be considered. Increasingly endothelial function testing is being explored for clinical management and evaluation of therapeutic response, although there are currently no guidelines recommending use of endothelial function in routine patient management. Nonetheless, evidence continues to grow in the role of the vascular endothelium in disease pathophysiology and ongoing large-scale studies are essential for the evaluation of therapies targeting endothelial function.

**TABLE 2:** Range of normal values for coronary flow reserve (invasive) and myocardial flow or perfusion reserve (non-invasive) with different imaging modalities.

Modality	Values used to diagnose CMD
**Invasive methods**	
Angiography + adenosine	CFR: abnormal <2.0 (94)
Angiography + acetylcholine	CFR: abnormal <1.5 (95)
**Non-invasive methods**	
CMR + adenosine	MFR: definite CMD <1.5, borderline CMD 1.5–2.6 (96) MPRI: abnormal <1.84 (97), ≤1.47 predicts MACE (98) Global stress MBF without visual perfusion defects: abnormal ≤2.25 ml/g/min (70)
PET + adenosine	MFR: definite CMD <1.5, borderline CMD 1.5–2.6 (96)
CT-perfusion	MFR: abnormal <2 (60)
Forearm plethysmography	No established cutoff
Finger plethysmography	RHI: <1.6–1.75 portends high risk for cardiovascular events (99)

*CMD, coronary microvascular disease; CFR, coronary flow reserve; MFR, myocardial flow reserve; MPRI, myocardial perfusion reserve index; MACE, major adverse cardiac events; MBF, myocardial blood flow; RHI, reactive hyperemic index. Non-invasive measures of MFR reflect CMD if significant contribution of reduced from epicardial coronaries has been ruled out.*

## Author Contributions

AM, EG, MC, AA-Z, SK, and AH drafted the manuscript. TL and AL edited the manuscript. All authors contributed to the article and approved the submitted version.

## Conflict of Interest

AA-Z has received research support from Canon Medical Systems. AL serves as a consultant for Itamar Medical. The remaining authors declare that the research was conducted in the absence of any commercial or financial relationships that could be construed as a potential conflict of interest.

## Publisher’s Note

All claims expressed in this article are solely those of the authors and do not necessarily represent those of their affiliated organizations, or those of the publisher, the editors and the reviewers. Any product that may be evaluated in this article, or claim that may be made by its manufacturer, is not guaranteed or endorsed by the publisher.
